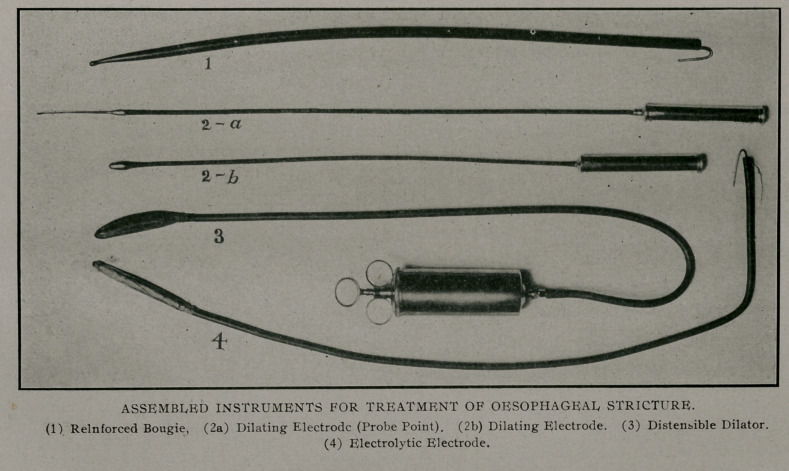# Treatment of Osophageal Stricture

**Published:** 1912-11

**Authors:** George C. Mizell

**Affiliations:** Atlanta, Ga.


### Journal-Record of Medicine

Successor to Atlanta Medical and Surgical Journal, Established 1855
and Southern Medical Record, Established, 1870.
OWNED BY THE ATLANTA MEDICAL JOURNAL CO.
**Published Monthly**

*Official Organ Fulton County Medical Society, State Examining Board, Presbyterian Hospital, Atlanta, Birmingham and Atlantic Railroad Surgeons' Association, Chattahoochee Valley Medical and Surgical Association, Etc.*

EDGAR G. BALLENGER., M. D., Editor.
BERNARD WOLFF, M. D., Supervising Editor.
A. W. STIRLING, M. D., C. M., D. P. H., J. S. HURT, B. Ph., M. D.
GEO. M. NILES, M. D., W. J. LOVE, M. D., (Ala.); Associate Editors.
E. W. ALLEN, Business Manager.
COLLABORATORS
Dr. W. F. WESTMORLAND, General Surgery.
F. W. McRAE, M. D., Abdominal Surgery.
H. F. HARRIS, M. D., Pathology and Bacteriology.
E. B. BLOCK, M. D., Diseases of the Nervous System.
MICHAEL HOKE, M. D., Orthopedic Surgery.
CYRUS W. STRICKLER, M. D., Legal Medicine and Medical Legislation.
E. C. DAVIS, A. B., M. D., Obstetrics.
E. G. JONES, A. B., M. D., Gynecology.
R. T. DORSEY, Jr., B. S. M. D., Medicine.
L. M. GAINES, A. B., M. D., Internal Medicine.
GEO. C. MIZELL, M. D., Diseases of the Stomach and Intestines.
L. B. CLARKE, M. D., Pediatrics.
EDGAR PAULIN, M. D., Opsonic Medicine.
THEODORE TOEPEL, M. D., Mechano Therapy.
R. R. DALY, M. D., Medical Society.
A. W. STIRLING, M. D., etc., Diseases of the Eye, Ear, Nose and Throat.
BERNARD WOLFF, M. D., Diseases of the Skin.
E. G. BALLENGER, M. D., Diseases of the Genito-Urinary Organs.

VOL. LIX. NOVEMBER 1912 No. 8

### TREATMENT OF OSOPHAGEAL STRICTURE.*

BY GEORGE C. MIZELL, ATLANTA, GA.

Contraction of the oseophagus is the most frequent of its pathological conditions. About 85% are malignant and the remaining 15% includes congenital constrictions, contraction of cicatrices, pressure from tumors, etc. Congenital stricture may be overlooked until the patient has reached manhood. Recently one such case, a young man, twenty-seven years of age, came for treatment. The history which he gave dated back to birth and no part of it was indicative of stricture. Because of supposed stomach trouble he had been on a soft or liquid diet

**Read before the Fulton County Medical Society.*

during his entire life. A type of stricture not often mentioned is one that is due to simple ulceration and cicatrical construction in or near the cardiac orifice.

We shall not speak of surgical cases and surgical measures for removal of new growths or those to provide a means of direct gastric feeding and the treatment of stricture from below. Such measures are necessary in those cases of stricture that are complete and those that will not admit of the passage of a small whalebone probe or the passage of a small steel olive electrode. Various methods may be employed for the diagnosis of stricture but the use of a probe and bougie is usually all that is necessary.

In all cases where a probe can be passed the treatment is chiefly mechanical. The mechanical treatment refrred to is the gradual dilation by means of safe and effective instruments. A number of instruments have been devised for this purpose, some of which are dangerous, others are difficult to manipulate.

We have here a collection of instruments (see plate) which are adapted to the treatment of any case in which mechanical dilation is indicated. These have been safe and effective in both simple and malignant stricture. When cancer is present the treatment can of course be only paliative and is directed to enabling the patient to take sufficient nourishment in a normal way to sustain life.

The treatment to be described is believed to be worthy of trial in any case in which a probe of any size can be passed. The steps of procedure depend and vary somewhat upon the size of the lumen of the stricture. Beginning with the largest bougie that will pass, a larger size should be passed each succeeding treatment until a number 30 can be introduced. In some cases this can be done with an English bougie, but it has been found that one which is relatively stiff can be more easily passed and is more safe. In using this instrument the force used must be gentle. This is especially true in malignant strictures and those due to corrosive substances. The danger here lies in rupture of the oesphagus and slipping of the mucus membrane with the shoulders of the instrument. Where the lumen will

ASSEMBLED INSTRUMENTS FOR TREATMENT OF OESOPHAGEAL STRICTURE.

(1) Relnforced Bougie, (2a) Dilating Electrodc (Probe Point). (2b) Dilating Electrode. (3) Distensible Dilator. (4) Electrolytic Electrode.

only admit the passage of a small whalebone probe or will not dilate from gentle pressure on the reinforced bougie (fig. 1) the next instrument to be mployed is a dilating electrode formed of the ordinary steel olive pointed dilator, the steel shank of which has been insulated with rubber tubing (fig 2-a & 2-b). To keep the instrument in the passage this electrode may have attached to the end a short whalebone probe (fig 2-a) This arrangement will admit of much more pressure than would otherwise be safe. Having passed the instrument into the oesophagus in the usual manner so that the olive is brought into contact with the obstruction a galvanic current is turned on; the negative pole being connected to the oesophageal electrode and the positive pole to another electrode in the shape of a belt which should in place under the arms before the oesophageal electrode is introduced. The current is now turned on and gradually increased up to 25 millimeters or until the olive has passed the stricture. The time taken for the current to reach the maximum should be about one minute. After the stricture has been passed the current should remain stationary until the instrument has been slowly withdrawn through the stricture. It should then be shut off while the instrument is being entirely withdrawn. Such treatment should be continued every day until the lumen will admit the introduction of a number 30 bougie. This instrument may be effectual and safe if continued to a greater degree of dilation but other methods are just as effectual and can be carried out with less discomfort to the patient and in most cases with a greater degree of results. From this point on the treatment is carried on with a distensible rubber and silk bag which has been placed over the lower end of a small stomach tube and an electrolytic electrode. The first instrument (fig. 3) from within is composed of, (1st) a brass spring wire stylet; (2nd) a small metal collar three-fourths of an inch long, and of such diameter that it will just fill the lumen of the stomach tube; (3rd) a small stomach tube; (4th) a rubber bag; (5th) a silk bag which should be strong and from three-fourths of an inch to one and one-fourth inches in diameter; (6th) a small rubber bag placed over the sik bag; this last bag serves to keep the silk bag clean and after being lubricated with vaseline facilitates introduction; (7th)

a glass or metal syringe for distending the bag. The assembled instrument should be tested for strength to withstand the maximum pressure to be employed and for leaks. The outside rubber bag should be replaced from time to time, as the grease rapidly destroys the rubber.

The electrolytic electrode (fig. 4) assembled consists, from within, of (1st), brass spring wire stylet; (2nd) conducting wire; (3rd) a small stomach tube; (4th) absorbent gauze covered with beta skin. The amount to gauze used should be enough to bring the size of the assembled instrument up to size of the largest bougie that has been passed.

Treatment with these instruments are given alternately every other day, or less frequently as may be indicated by the progress.

The purpose of this electrode is through electrolysis, to deposit thiosinamin in the tissues and thereby promote absorption of scar tissue while with the steel electrode the current serves to relax and emulsify the tissues.

Having dilated the stricture with the steel electrode or bougie to where it will admit a small rubber dilator, and the electrolytic electrode treatment is continued in the following manner:

The dilating instrument in the collapsed state is passed into the oesophagus so that the bag rests within the constriction. The stylet is now withdrawn and the bag distended by forcing water or air into the tube from the syringe.

The pressure should be maintained for about a minute. Before removing the instrument it should be deflated by withdrawing the water or air with the syringe, as this will render the removal of the instrument more comfortable to the patient. A small silk bag should be used about two weeks and a larger one substituted at the end of every such period. On every third day the electrolytic electrode should be used. The gauze is saturated with a solution of three grains of theosinamin in water and introduced with the stylet in place after the usual manner of introducing a bougie. The stylet is then withdrawn and the conducting wire is connected to the positive pole of a galvanic controller. The negative pole being connected to a belt electrode strapped around the body just below the arms. The current is gradually turned on until the register shows ten millimeters, or stopping just short of pain. This current is continued ten minutes, then it is reversed, that is, the osophagel electrode is changed to the negative pole for one minute. This change serves to relax the tissues so that the electrode can be easily withdrawn.

In malignant stricture the degree of dilation attempted is not as great as in non-malignant cases, and greater precautions are necessary. In these cases a lumen that will admit a 30 to 34 bougie will serve to enable the patient to take sufficient nourishment. When this degree of dilation has been attained a bougie should be passed every few days to preserve the opening. The patient may be instructed to introduce the bougie himself, which he may learn to do with very little discomfort.

In the treatment of simple strictures the size of the inflating instrument should be increased until the bag of one and one-fourth inches in diameter has been used.

Contraction following the swallowing of corrosive substances may involve the whole length of the tube but usually it involves only the lower end and the region opposite the cricoid cartiage. In such strictures it may be necessary to treat the contracted parts in segments.

## Figures and Tables

**Figure f1:**